# Barriers to treatment adherence among patients with tuberculosis: a qualitative study of Pakistani nationals and Afghan refugees

**DOI:** 10.1136/bmjopen-2025-100882

**Published:** 2025-10-05

**Authors:** Noor Sanauddin, Fayaz Ahmad, Maryiam Rahim, Muhammad Awais Paracha, Zohaib Khan, Fatima Khalid Qazi, Shaista Rasool, Mirrat Butt, Farooq Naeem, Muhammad Firaz Khan, Saima Sheikh, Zeeshan Kibria, Nishani Fonseka, Mian Ul-Haq, Saeed Farooq

**Affiliations:** 1 Department of Sociology, University of Peshawar, Peshawar, Khyber Pakhtunkhwa, Pakistan; 2 Khyber Medical University, Peshawar, Khyber Pakhtunkhwa, Pakistan; 3 Mayo Hospital, Lahore, Punjab, Pakistan; 4 Psychiatry, Queens University of Charlotte, Charlotte, North Carolina, USA; 5 School of Medicine, Keele University, Keele, Staffordshire, UK; 6 Lady Reading Hospital, Peshawar, Khyber Pakhtunkhwa, Pakistan

**Keywords:** Refugees, Tuberculosis, Medication Adherence, MENTAL HEALTH

## Abstract

**Abstract:**

**Objectives:**

Non-adherence to tuberculosis (TB) treatment remains a major challenge in high-burden regions. However, few studies have qualitatively examined the sociocultural and emotional barriers to adherence, particularly among Afghan refugees in Pakistan. This study explores the patient-related, sociocultural and treatment-related barriers to treatment adherence among patients with TB of Pakistani and Afghan origin living in Pakistan.

**Design:**

We conducted an exploratory qualitative study consisting of semistructured focus group discussions (FGDs) and in-depth interviews (IDIs) with purposively selected multisectoral stakeholders. The data were analysed thematically using a combination of inductive and deductive approaches.

**Settings:**

We employed a qualitative study design in the TB DOTS (Directly Observed Treatment Short course) centres in the Haripur and Peshawar districts of Khyber Pakhtunkhwa province, Pakistan.

**Participants:**

We conducted IDIs (n=29) and FGDs (n=11) with three categories of participants: TB healthcare providers, patients with TB and their carers.

**Results:**

We identified several contributors to lower treatment adherence. These included patient-related barriers (eg, lack of awareness about TB and its treatment), sociocultural barriers (eg, stigma, refugee status of Afghan patients, gender roles and reliance on traditional and spiritual healing) and treatment-related barriers (eg, demanding treatment regimen and TB-induced depression).

**Conclusion:**

Several personal, sociocultural and treatment-related barriers contribute to lower treatment adherence in patients with TB. A significant contributing factor to treatment non-adherence in patients is the high prevalence of anxiety and depression related to TB and its treatment, for which there is no treatment or counselling available at the DOTS level in Pakistan, warranting the need for mental health interventions that could improve adherence and treatment outcomes for both TB and depression.

**Trial registration number:**

ISRCTN10761003.

**STRENGTHS AND LIMITATIONS OF THIS STUDY**

- A rigorous qualitative design was employed that is, a combination of in-depth interviews and focus group discussions in the study for a comprehensive overview of the patient-related, sociocultural, as well as treatment-related barriers which negatively influence the adherence to tuberculosis (TB) treatment.
- Diverse stakeholder groups, including healthcare providers, patients with TB and carers, were included to provide a broad range of the challenges that affect treatment adherence from various perspectives.
- As the study is based on self-reported data, the possibility of recall bias and social desirability bias is particularly high when discussing stigmatising topics (TB or mental health).
- The study sheds light on the unique challenges faced by Afghan patients, particularly those with undocumented refugee status in Pakistan, in accessing healthcare services, including TB treatment, underscoring the need for targeted interventions to ensure equitable access to care.
- Given that the study was conducted in specific areas of Pakistan (Peshawar and Haripur) may not generalise to other regions of Pakistan and Afghanistan.

## Introduction

 Tuberculosis (TB) is a significant public health threat worldwide and is the leading cause of death due to a single infectious agent.^1^ Globally, an estimated 10.6 million people suffered from TB, causing the deaths of 1.6 million in 2021.^2^ A systematic review found that almost 25% of the world’s population could be infected by TB.^3^ Low- and middle-income countries (LMICs) bear the highest disease burden. Pakistan, an LMIC with a population of over 245 million, ranks fifth among the TB high-burden countries, with the fourth highest prevalence of drug-resistant TB (DR-TB) globally and an estimated half a million new cases of TB each year.^1^

Several factors contribute to this high burden of TB in LMICs, low adherence to TB treatment being the foremost, which results in prolonged disease transmission and resistance to anti-TB drugs.^4^ A systematic review found that the proportion of patients who initiate treatment ranges from 24% to 98%, while those completing treatment vary from 19% to 90%.^5^ A study in South Africa estimated that only 53% of overall TB cases completed their treatment,^6^ while a cohort study from Colombia found only 38.5% treatment completion.^7^ The rate of non-adherence to TB treatment is found to be 30% in Nigeria.^8^ Another study found that 67.3% of treatment defaults occurred within the first month.^9^ In Pakistan, the prevalence of non-adherence to TB treatment, according to the Morisky medicine adherence scale, was found to be low adherence in 44% and medium adherence in 33% of patients with TB.^10^ A recent study in Afghanistan found a 66.8% non-adherence rate.^11^

The factors influencing adherence vary from country to country mainly because the factors are usually sociocultural. Second, a country’s sociodemographic and economic conditions affect treatment adherence. In Ghana, for example, where poverty and illiteracy are matters of concern, the primary factors associated with low adherence to treatment in patients with TB include transportation costs, difficulty in accessing public transport, low income and lack of knowledge about TB.^12^ In the Indian subcontinent (India, Pakistan, Nepal and Bangladesh), personal influences such as a role in the family, daily routine activities, the experience of the illness, the interaction of healthcare providers with patients and other structural, social, economic and cultural factors influence treatment adherence.^13^

Some studies have focused on health system-related factors, such as the lack of trained healthcare providers, inefficient application of treatment protocols and insufficient supervision by senior healthcare providers as barriers to treatment adherence.^14 15^ TB-related stigma has also been identified as a significant barrier to adherence.^16^ A critical finding of many studies is that mental health as a comorbidity among patients with TB is a barrier to treatment adherence. A recent systematic review found a close association between depression, poor adherence and loss to follow-up (LTFU).^17^ A study from India found a 55% prevalence of depression among patients with TB, and 41.8% of depressed patients had poor treatment adherence.^18^ Studies from China report that depression is closely associated with poor treatment adherence and low quality of life among patients with TB^19^ and that depression and disease-related stigma are barriers to treatment adherence.^20^

Incomplete and inadequate treatment may lead to prolonged infectiousness, multidrug-resistant TB, increased mortality and healthcare expenditure.^21^ Therefore, addressing non-adherence to TB medications is an urgent need that requires a more profound and comprehensive understanding of the barriers to TB treatment adherence for better outcomes.

Only a few studies conducted in Pakistan have qualitatively explored the sociocultural determinants of adherence to anti-TB therapy. An earlier qualitative study conducted in Karachi found that barriers to treatment adherence included poor knowledge about the disease, lack of adequate counselling by health personnel, low financial status and distant health facilities.^22^ A recent study has highlighted factors behind LTFU among patients with DR-TB in Karachi and found that patients’ perceptions about DR-TB treatment, socioeconomic condition, medication and service provider-related factors are significant barriers to the successful completion of treatment.^23^ These studies are limited to Karachi, a metropolitan city with entirely different sociodemographic characteristics from this study’s settings. Second, these studies have explored only the patients’ perspectives, leaving the views of carers and healthcare providers unexplored.

In Afghanistan, however, no qualitative study has been conducted to identify the barriers to treatment adherence among patients with TB, nor has any study covered Afghan refugees living in Pakistan or elsewhere in the world to explore how their sociocultural background and refugee status might influence treatment adherence. We aimed to fill this gap and provide insights into the unique challenges faced by the Pakistani and Afghan refugee population in accessing and adhering to TB treatment, exploring the perspectives of patients, carers and healthcare providers.

## Methodology

### Study design

This study used qualitative methods to understand the complex personal, sociocultural and treatment-related barriers affecting TB treatment adherence. Data were collected from various stakeholders, including healthcare providers, patients with TB and carers, through in-depth interviews (IDIs) and focus group discussions (FGDs).

### Study setting

The study was conducted in DOTS (Directly Observed Treatment Short-course) centres of Pakistan’s National TB Control Programme (NTP). The NTP delivers services for DR-TB and drug-sensitive TB through the DOTS centres, also called basic management units (BMUs), located within primary, secondary and tertiary healthcare facilities in every district of Pakistan. We purposively selected two districts in the Khyber Pakhtunkhwa (KP) province of Pakistan—Peshawar and Haripur—based on the TB burden and the presence of the Afghan refugee population in these districts. The KP province is located in the northwestern region of Pakistan, which shares a 2600 km porous border with Afghanistan. People on both sides of the border share the same ethnic identities, allowing them to maintain close social and cultural ties with frequent crossovers for business and social purposes. According to the latest census, KP province has the largest rural population, with nearly 85% of its total 40.85 million people living in rural areas.^24^ KP province hosts the largest number of the estimated 3.7 million Afghan refugees living in Pakistan. Among these, only 1.3 million are registered Afghan refugees, while the rest either hold only Afghan Citizenship Cards or are undocumented.^25 26^ Most of the local population of the province, as well as the refugees, belongs to the Pashtun ethnic group who speak the Pashto language. Hindko is the second most spoken language of the province, primarily spoken in the Hazara regions, including the Haripur district. Both Peshawar and Haripur have a high number of refugees, and they have limited access to health and other social services. The literacy rate of KP is 55.1%, with a considerable gender gap (37.4% for women and 72.8% for men).^27^ This gap is particularly prominent in the Pashto-speaking rural areas, where traditional gender norms have generally limited the education of women.

### Sampling and sample size

Qualitative research does not aim to seek representative findings; therefore, in line with good practice recommendations for qualitative research, a non-probability sampling approach was adopted to purposively select participants for the study.

We recruited three groups of participants: (1) patients with TB, (2) carers of the patients and (3) healthcare providers at different TB DOTS centres in the two districts. As shown in table 1, we conducted 29 semistructured interviews (16 in Peshawar and 13 in Haripur) and 11 FGDs (7 in Peshawar and 4 in Haripur). Both IDIs and FGDs are helpful research techniques, and by triangulating the methods and participants, we aimed to gather rich information from various sources. While the interview is better suited for getting in-depth information from an individual about their personal beliefs and experiences, FGDs help reveal group norms and sociocultural values, which influence the behaviour of people. We determined that data saturation was achieved when no new themes, concepts or codes emerged from the interviews. This occurred after 29 IDIs and 11 FGDs, at which point additional data no longer contributed to new analytical insights. Saturation was assessed through ongoing team discussions and comparison of coded data.

**Table 1:** Number of FGDs and IDIs conducted by participant type and nationality

Categories of participants		District Peshawar		District Haripur	
		FGDs	IDIs	FGDs	IDIs
Patients	Pakistani	02	03	02	03
	Afghan	01	04	01	02
Carers	Pakistani	02	02	01	03
	Afghan	01	04	Nil	01
Healthcare professionals		01	03	Nil	04
**Total**		07	16	04	13

Each FGD contained 6–7 participants (see table 3).

FGDs, focus group discussions; IDIs, in-depth interviews.

Separate FGDs were conducted with healthcare providers, male and female patients and carers. The healthcare providers included DOTS facilitators and medical officers, as every BMU comprises at least one person in each position. The research team identified eligible participants with the help of DOTS facilitators who play a crucial role in the BMU, as they are the first point of contact for patients with TB. Patients with TB who had completed at least 1 month of TB treatment were recruited. Similarly, a relative of the patients involved as the primary carer and who accompanied the patients to the BMU was eligible to be recruited. To ensure maximum variation in the sampling, we recruited participants of both genders, ages (20–64 years), ethnicities (Pashtun and Hazara) and nationalities (Pakistani and Afghan) as shown in table 1. None of the participants refused to participate in the study.

Throughout the data collection, the research team engaged in daily debriefing sessions with the data collection team, where we discussed emerging themes, methodological and practical challenges and the necessity for additional data collection. These iterative sessions enabled us to reflectively assess our recruitment strategy and determine whether further interviews or focus groups would yield novel insights. Given the substantial sample size of this study, our team collectively determined that continued data collection would likely result in diminishing returns, thereby indicating saturation. Consequently, we ceased data collection, confident that our dataset was sufficiently comprehensive to address our research objectives.

### Data collection

Research assistants with public health backgrounds, trained in ethical and unbiased data collection, gathered data from January to March 2023. All interviews and FGDs were conducted in person by the qualitative research team in a vacant office or room at the DOTS centre that was convenient for the patient with attention to gender sensitivity, transparency and participant autonomy. Separate topic guides were developed for each group of participants, which were translated into local languages (Urdu and Pashto). The topic guides covered the lived experiences of patients with TB and carers, as well as their personal and sociocultural circumstances and treatment-related perceptions and experiences which might influence patients’ adherence to TB treatment. We pilot-tested the topic guides in both districts and used the refined versions for data collection. Each FGD lasted 50–70 min, while the IDIs lasted 35–45 min. The IDIs and FGDs were conducted in either Pashto or Urdu as per the study site or the background of the participants and were audio-recorded. Filed notes were taken after the interviews and FGDs. A written informed consent was obtained from all participants after explaining the purpose of the study (and establishing relationship). Participants were invited to the local health facilities for interviews and FGDs. We gave each participant a cash payment (2000 PKR, equivalent to £6) for travel costs. Reimbursement for travel costs incurred in attending the interview and FGDs at local health facilities. This amount was determined based on average local transport expenses to ensure that participation in the study did not place a financial burden on the individuals involved. This incentive aimed to reduce financial barriers that might otherwise prevent participation, particularly among individuals from socioeconomically disadvantaged backgrounds. The amount was carefully determined to avoid any form of coercion or undue influence and was approved by the ethics committee (Keele and Khyber Medical University) as part of the study protocol.

### Data analysis

All the interviews were transcribed verbatim, translated into English, anonymised and proofread against the audio recording by the research team. Data were analysed using the framework analysis approach, which allows deductive and inductive techniques. First, we thematically analysed five transcripts to identify significant themes. After this, we developed a thematic framework in MS Excel based on the identified themes, the research questions and topic guides. Later, all the transcripts were subjected to the thematic framework, focusing on exploring the patient-related, sociocultural and treatment-related factors acting as barriers to treatment adherence among patients with TB. The themes and subthemes identified within the data were charted in the framework with verbatim quotes. The charted data were reviewed and analysed to compare views, seeking patterns, connections and explanations. After several iterations and cross-checks of data among the analysis team members, summaries of findings were finalised for each participant group. To ensure analytical validity, we employed multiple coders to independently code the data. We also engaged in peer debriefing, where colleagues reviewed and provided feedback on our analysis, to identify potential biases and improve the validity of our findings. These steps helped to increase our confidence in the accuracy and reliability of our results. In addition, to enhance the validity and reliability of our findings, we used a data triangulation approach, where multiple data sources were leveraged to corroborate and validate our results. Specifically, we integrated data from interviews and focus groups to facilitate a comprehensive understanding of the phenomenon under investigation. The triangulation of data sources enabled us to verify the consistency of our findings across different methodological approaches, thereby augmenting the accuracy and reliability of our results. Furthermore, this approach allowed us to capture a more nuanced and multifaceted understanding of the phenomenon, thereby enriching our insights. Two senior research team members (NS and FA) supervised the data analysis. Further details regarding researcher reflexivity and positionality throughout data collection are provided in the Reflexivity Statement (online supplemental file).

### Patient and public involvement

Patient and public involvement and engagement (PPIE) was an integral part of this project, embedded throughout all stages of the research. Before commencing the qualitative fieldwork, we held a dedicated PPIE meeting with a service user group comprising both patients and healthcare providers. During this session, we shared our draft topic guides and received valuable feedback, which was carefully considered and incorporated into the final tools. The group highlighted important issues such as the need for gender sensitivity in data collection, recommending that female participants be interviewed by female researchers and advised that FGDs be conducted within hospital settings to ensure comfort and accessibility for participants. These contributions significantly strengthened the cultural and contextual appropriateness of our study design and fieldwork procedures.^28^

## Results

### Sociodemographic characteristics of study participants

Table 2 presents the distribution of participants by method, and gender, and number of participants along with their mean ages. Table 3 represents site (Peshawar/Haripur) and demographics of individual participants within each ID and FGD. In Peshawar, 46 participants took part in FGDs and IDIs, with men slightly outnumbering women. Male participants had a mean age ranging from 43.7 (FGDs) to 45.4 years (IDIs), while female participants ranged from 39.6 to 42.8 years. In Haripur, 39 participants were included, with similar numbers of men and women across methods. The mean age of Haripur participants varied more, with male participants ranging from 39.3 (IDIs) to 43.3 years (FGDs), and female participants from 40.0 (FGDs) to 47.0 years (IDIs). Overall, male participants were generally older in Peshawar, while the oldest average age was among women in Haripur IDIs.

**Table 2:** Demographics of individual participants within each IDI and FGD

Characteristics		Peshawar				Haripur			
		FGDs		IDIs		FGDs		IDIs	
		M	F	M	F	M	F	M	F
No. of participants		33	13	9	7	12	14	6	7
Nationality	Pakistani	20	13	5	3	6	14	4	6
	Afghan	13	0	4	4	6	0	2	1
Type of participant	Patients	15	6	3	4	12	6	3	2
	Carers	13	6	3	3	0	8	2	2
	Health professionals	5	1	3	0	0	0	1	3
Employment status	Employed	30	2	7	0	8	3	6	4
	Unemployed	3	11	2	7	4	11	0	3

FGDs, focus group discussions; IDIs, in-depth interviews.

**Table 3:** Age and gender distribution of participants

Site	Method	Gender	No. of participants	Mean age
Peshawar	FGDs	Male	33	43.7
		Female	13	42.8
	IDIs	Male	9	45.4
		Female	7	39.6
Haripur	FGDs	Male	12	43.3
		Female	14	40.0
	IDIs	Male	6	39.3
		Female	7	47.0

FGDs, focus group discussions; IDIs, in-depth interviews.

The data include participants from Peshawar and Haripur across FGDs and IDIs, with more participants in Peshawar (75) than Haripur (39). Most participants were Pakistani, while Afghan participants were mostly men, especially in Peshawar. Patients formed the largest group overall, with carers mainly in Peshawar and health professionals mostly in Peshawar male groups. Male participants were largely employed, whereas most female participants—particularly in FGDs—were unemployed. Haripur had higher female participation, while Peshawar had a more diverse mix, including a higher proportion of Afghan nationals and health professionals.

### Barriers to TB Treatment

We identified several personal/patient-related, sociocultural and treatment-related themes and subthemes contributing to poor treatment adherence among patients with TB, described in the following section.

#### Patient-related factors

Several patient-related subthemes that contribute to poor adherence to TB treatment were identified, and the most important and frequently reported by participants are detailed below.

##### Inadequate knowledge among patients about TB and the importance of treatment

Healthcare professionals (DOTS facilitators) identified the lack of knowledge and awareness among patients and carers as the primary barrier to treatment adherence. They reported facing significant challenges in conveying the importance of taking medication on time and ensuring patients understand the importance of not missing doses. It was revealed that instead of following the doctor’s advice, some patients take medicines based on their perceived wellness and illness, which leads to drug resistance.

The most important factor is awareness; patients are not properly aware [about TB and its treatment]. This may be due to us, our staff (at DOTS), and doctors. People do not know the importance of treatment adherence; they take medicine for a few days then leave it thinking that he/she has got better*.* (IDI-Peshawar-Health Professional)

While some patients reported reducing their medication dosage because they started feeling better, others shared that they had been taking multiple medications for a long period without seeing any improvement in their health, causing them to stop taking their medicines. The quotes from healthcare professionals and carers underscore the need for improved patient education, awareness and support to promote treatment adherence and address the challenges posed by misconceptions and cultural beliefs about TB.

TB treatment is a long course; when they observe improvement in their health, it leads to satisfaction, so they stop taking medicines. (FGD-Peshawar-Health Professional)

Additionally, it was found that some patients appeared to have little trust and confidence in TB medicines. Some Afghan nationals also believed that TB is a divine affliction and not curable entirely through medication. As a result, such patients hesitate to consult medical professionals, often delaying the registration of patients at DOTS and causing non-adherence to medication once treatment is started.

It was also shared by some carers that sometimes their patients refuse to take preventive measures and sometimes lie about taking medicine to avoid them. Carers further shared that they cannot be strict with their patients and cannot force them to take medicines.

Sometimes he (the patient) refuses to take the medicines altogether and sometimes lies about it. (IDI-Haripur-Pak-Male-Carer)

The lack of knowledge and awareness about TB and its treatment among patients and carers is a significant barrier to treatment adherence, with patients often taking medicines based on perceived wellness rather than following the doctor’s advice, leading to drug resistance. Additionally, some patients’ limited trust in TB medicines, cultural beliefs (eg, TB being a divine affliction) and refusal to take preventive measures further contribute to delayed registration at DOTS and non-adherence to medication.

##### Multimorbidity and frailty in old age patients

Our findings reveal that older patients lack an understanding of their disease, and their physical frailty contributes to treatment failure. It was mentioned that older patients are more likely to have comorbid conditions, which could potentially lead to treatment default. They get weaker, and it reduces their capability to complete treatment.

The older a patient, the adverse effects of symptoms. Some elderly patients do not even acknowledge that they have TB*.* (IDI-Peshawar-Health Professional)

It has been three months since his treatment started. He is also a cardiac patient. He is at the age of 75 and frail. (IDI-Peshawar-Pak-Male-Carer)

Sometimes for elderly patients other patient-related factors such as forgetting to take medication on time or catching up on a follow-up appointment also contribute to their vulnerability. In such cases, the role of family was critical in helping the patients adhere to medication and treatment regimens.

Older patients with TB face unique challenges, including physical frailty, comorbid conditions and limited understanding of their disease, which can contribute to treatment failure and default. The role of family support is critical in helping older patients adhere to medication and treatment regimens, particularly when patients experience cognitive or physical limitations that affect their ability to manage their treatment. By acknowledging these factors, healthcare providers can develop targeted strategies to support older patients and improve their treatment outcomes.

### Sociocultural factors

The social and cultural factors influencing adherence to TB treatment identified in this study include perceived stigma, gender issues, poverty and reliance on traditional medicines.

#### Social stigma related to TB

Patients suffering from TB experience stigma, which affects their personal, social and mental well-being, leading to poor treatment adherence. Participants mentioned that people avoid contact with patients with TB and discriminate against them, leading to their social isolation and mental health deterioration.

In the whole neighbourhood, only I have TB. Everyone thinks of me as a dead body that has been dug out and is alive again: ‘laka yao marhay che da gor na robasi o bya e jawanday ki’. (IDI-Peshawar-Pak-Male-Patient)

Some patients even reported being treated with contempt by their family members.

My aunt feels disgusted (‘krak jana’) and stays away from me. (FGD-Peshawar-Pak-Female-Patient)

The stigma surrounding TB leads patients to hide their illness, resulting in low adherence to medication, or, in some cases, patients avoid seeking treatment altogether due to fear of stigma.

I don’t know, but I want to cry when I look at myself. People don’t even know about my illness; still, they ask why my colour is becoming dull day by day. So, I wonder what they will say if they find out that I have TB*.* (FGD-Haripur-Pak-Female-Patient)

The stigma surrounding TB is deeply rooted in cultural and social norms, which leads to social isolation, mental health deterioration and poor treatment adherence. The quotes from patients highlight the emotional pain and distress caused by stigma, which can be as debilitating as the physical symptoms of the disease. The stigma surrounding TB is not just a personal issue but also a public health concern, as it can lead to poor treatment adherence and increased transmission of the disease. By addressing TB-related stigma, we can improve treatment adherence, reduce social isolation and promote the overall well-being of patients with TB.

#### Gender issues: women patients’ responsibility to take care of children and restrictions on their movement

The patriarchal norms in the region require women to be accompanied by male relatives whenever they go out of the home. This appeared as a significant barrier to getting timely treatment and reduced adherence to medication for female patients. In most cases, female patients must wait until a male family member is ready to accompany them to the DOTS centre. Some married female patients also pointed out that they sometimes miss follow-up appointments because they cannot leave their little children (and other dependents) at home.

… and when the prescription finishes, who will refill my medicine? … I am alone, a female, I cannot fetch them, and there is no other (male) adult in the house (to fetch medicines or accompany me)*.* (IDI-Haripur-Pak-Female-Patient)

It is a widespread belief in the Pashtun culture that women often use illness as an excuse to avoid household chores or to seek the attention of their husbands and other family members. In a particular case, the husband of a patient showed no support and left her at her mother’s house, claiming that she was not sick and was just pretending.

We are currently treating a married woman. According to her husband, she is making excuses and that she is completely all right. … … (Her husband says that) until she is completely cured, she will stay with her mother*.* (FGD-Peshawar- Health Professional)

The quote from the health professional highlights the lack of support from family members, particularly husbands, in recognising and addressing women’s health needs. This can lead to women feeling isolated, unsupported and struggling to manage their condition. Women patients find it more challenging to follow treatment regimens because of cultural factors limiting their decision-making powers about health and well-being. The requirement for women to be accompanied by male relatives when going out of the home can significantly delay their access to healthcare services, including TB treatment. Similarly, women patients’ responsibility to take care of children and dependants can make it challenging for them to prioritise their own health needs. This can lead to missed appointments, delayed diagnoses and poor treatment adherence. The widespread belief that women use illness as an excuse to avoid household chores or seek attention can contribute to the stigma and lack of understanding around women’s health issues. In short, the findings of this study highlighted the power dynamics at play in patriarchal society, where women’s decision-making powers about their health and well-being are limited. This can lead to women being unable to prioritise their own health needs, adhere to treatment regimens or seek healthcare services without permission from their male family members.

#### Poverty

Almost all patients and their carers referred to their financial hardships directly or indirectly, explaining how their worries about meeting their living expenses set aside their health needs as secondary. Although TB medications are provided free of cost at the DOTS facilities, other related expenditures like investigations and diagnosis contribute to non-adherence to treatment. DOTS facilitators explained that sometimes specific diagnostic tests are unavailable at the facility, and patients must undergo these investigations in a private setting, which is an out-of-pocket expenditure.

Even GeneXpert is not performed at all Tertiary Care Hospitals For X-rays, patients need to pay, which they cannot afford*.* (IDI-Peshawar-Health Professional)

In some cases, patients must purchase medicines from the private market, which also incurs a substantial cost.

Furthermore, poverty was also mentioned as the main factor leading to stress and anxiety among patients, which contributes to low adherence to the treatment. Healthcare providers also mentioned that poor people are more concerned about the economic needs of their families than their own health.

We repeatedly tell them to take care of their food and improve their lifestyle, but they still prioritise their financial state over their health*.* (IDI-Peshawar-Health Professional)

TB can have a significant impact on a person’s ability to earn income, which can exacerbate their financial struggles and push them further into poverty. This creates a cycle where poverty and TB reinforce each other. Some patients in this study elaborated on how they lost their jobs or saw a decrease in their income, due to which they found it challenging to meet the expenses related to TB treatment. An Afghan female patient who used to be a tailor expressed how TB has pushed her into poverty.

Now I do not earn; previously I used to earn Rs. 7000-8000 per month. I used to receive orders of clothes and sew them. And now in this disease, I am helpless*.* (IDI-Peshawar-Afghan-Female-Patient)

Some of the caregivers expressed that due to poverty, they were unable to provide patients with the diet prescribed by doctors and had to restrict them to the available and affordable food. This issue was more pronounced in a joint family system where patients had to eat whatever was available for other family members.

The doctor advised us to give her good food (fruits), but we cannot afford it. So, we give her whatever we can afford. (FGD-Peshawar-Afghan-Male-Carer)

Many patients associate their depressive symptoms with their weak financial position, especially those who are the sole earners of their families. Apart from the expenses related to treatment, TB can result in the loss of jobs and negatively influence the earning capacity of the patients. Moreover, most patients lived in a joint family system and were worried about the transmission of infection to other family members.

The most evident reason which makes one depressed after getting the disease is the thought that ‘I can’t recover fully like before’. Second, a major source of depression is poverty; the patients might find that they can’t afford treatment. Taking loans and taking care of children and elders in the joint family system, etc., create a lot of problems and stress for the poor patients*.* (FGD-Peshawar-Afghan-Male-Patient)

Financial hardships significantly contribute to non-adherence to TB treatment, as patients struggle to afford related expenditures while also facing reduced income and increased stress due to the disease’s impact on their earning capacity, creating a cycle where poverty and TB reinforce each other.

#### Issue of accessibility and transportation to the healthcare facility

Access to healthcare facilities also influences patients' treatment routines. Several participants pointed out that the DOTS centre is not easily accessible, and some patients from remote areas had to travel long distances. Geographical scatteredness, vast catchment areas of a DOT facility, a poor public transport system and the high cost of private transportation decrease patients’ access to DOT centres.

Yes, medicines are free, but transportation requires money, and there is inflation nowadays, too. We are poor people, and there is no specific income source*.* (IDI-Peshawar-Pak-Male-Patient)

Patients and carers mentioned spending substantial money on transportation for treatment and follow-up. Travel fatigue and transportation charges burden the patients’ pockets and elevate their stress levels. The accessibility issues were more severe for female patients, who, as explained elsewhere in the paper, cannot travel alone without the company of a male relative.

I used to travel to Pakistan from Afghanistan…. I used to spend almost Rs 5000/- each time for transport only. My young children would collect scrap, and we collect money (to visit the doctor) by selling scrap …. (IDI-Peshawar-Afghan-Female-Patient)

I also have transport issues. We have to transfer between two vehicles to get here…. The taxi fare ends up being more than the costs of my medicine. (IDI-Peshawar-Pak-Male-Patient)

Afghan patients reported experiencing significant challenges in accessing TB treatment in Pakistan, largely due to their status as Afghan nationals or refugees living in Pakistan.

Overall, (getting) treatment was difficult for me because I had no (Proof of Registration) Card, and I had to visit for 15 days consecutively to get medicines as I have no Card. (FGD-Peshawar-Afghan-Male-Patient)

Another problem with Afghan refugee patients was frequent relocation. The patients sometimes go to Afghanistan and come back after a break. A healthcare provider explained that sometimes registered Afghan refugees go to Afghanistan and fail to show up for an appointment within one or 2 months, leading to their classification as ‘defaulters’.

These Afghan patients sometimes leave for Afghanistan after a month or two. This is how they are counted as defaulters… or sometimes they shift their place inside Pakistan. (IDI-Haripur-Health Professional).

A female Afghan patient shared that her illness remained undiagnosed for a long time and that she had to commute between Pakistan and Afghanistan for her TB treatment, resulting in spending a considerable amount of money on travel.

I used to bear this pain for 03 years. No doctor (in Afghanistan) could understand my treatment. I visited many doctors. …. Then, I travel to Pakistan from Afghanistan and started my treatment here. (IDI-Peshawar-Afghan-Female-Patient)

#### Relying on traditional, alternative treatment

Patients relied heavily on traditional and alternative medicines, which most often compromise compliance with recommended treatment. Older people in Pakistan and Afghanistan usually have more faith in *hakim* (herbalists) for the treatment of diseases, including TB. The reliance on traditional medicine can lead to delayed diagnosis and treatment of TB. Patients may try various traditional remedies before seeking medical attention from a healthcare provider, which can worsen their condition and reduce the effectiveness of treatment.

Before coming to the TB facility, I took my mother to Hakim Kirdaani (herbalist). Someone recommended to my mother that she would get healed from illness if she got treatment from that hakim*.* (FGD-Peshawar-Afghan-Male-carer)

Several patients acknowledged having tried traditional medicines and *‘totkay’* (traditional tips or home remedies) before visiting a doctor.

Many old people come here with TB who happen to treat their symptoms first at home by taking various kinds of traditional remedies, and when they fail to get well, they visit us*.* (IDI-Haripur-Health Professional)

The reliance on traditional medicine among patients in Pakistan and Afghanistan reflects the complex interplay between cultural, social and economic factors that influence treatment-seeking behaviour. Some potential factors contributing to this reliance include limited access to healthcare, leading patients to seek alternative forms of treatment. Traditional medicine is deeply ingrained in the cultural and social fabric of these countries, making it a preferred option for many patients. Lastly, patients may not be aware of the effectiveness of modern medicine in treating TB, or they may not understand the risks associated with traditional remedies.

The sociocultural barriers highlighted in this section, such as social stigma, gender discrimination, poverty, refugees’ status of Afghan patients, poverty-induced depression and reliance on traditional medicines, play a significant role in non-compliance with treatment among patients with TB. Some health professionals also noted a steady decrease in the number of patients who registered at DOTS over time and attributed this decrease to poverty as people cannot afford treatment.

### Treatment-related factors

Treatment of TB requires commitment, and many patients struggle to comply with the demanding regimen. The subthemes concerning treatment-related barriers identified in this study include heavy doses, longer duration, side effects of TB treatment and mental health comorbidities, especially depression among patients with TB.

#### Difficult treatment routine and heavy doses

Patients and carers reported challenges following the doctor’s suggestions regarding the medicines’ frequency, time and dose size. For many patients, taking medicines without the assistance of someone in the family was a troublesome job. Participants mentioned that issues were more noticeable in the initial days of the treatment as most patients found it challenging to swallow ‘large tablets’ due to their ‘bitter taste’.

Most patients get scared even after seeing the (large) pack of medicines. When my friends saw it, they said, ‘Oh, you will eat all this in a month?’ (IDI-Haripur-Pak-Male-Patient)

I also find taking medicine very hard. They are bigger in size, and they often stuck in my throat. (FGD-Haripur-Pak-Female-Patient)

Healthcare providers also mentioned that sometimes patients complain about swallowing and digesting the tablets.

Thus, it was found that patients and carers face challenges in adhering to TB treatment regimens, particularly in the initial stages, due to difficulties in swallowing large tablets with bitter taste, highlighting the need for support and guidance from family members or healthcare providers to overcome these obstacles and ensure successful treatment outcomes.

#### Long duration of TB treatment

According to participants, the long duration of TB treatment also contributes to non-adherence. The treatment for TB typically lasts for 6–8 months, which can be quite challenging for patients as well as their families. Patients explained that visiting the DOTS centre repeatedly for extended periods is difficult, putting a significant physical, emotional and financial burden on patients and carers. Healthcare providers explained that some patients become hopeless due to the slow progress in treatment, which leads to depression among patients.

It is difficult to take medicines continuously for six months. It is too long. Some people leave treatment because when they take medicines for such a prolonged duration, they feel sad, weak, and annoyed with TB drugs*.* (FGD-Haripur-Pak-Male-Patient)

Participants also referred to the psychological effects of the longer duration of treatment.

Further, the treatment is not for days; it is for months. … the patient gets worried, which leads to depression*.* (IDI-Peshawar-Health Professional)

The long duration of TB treatment contributes to non-adherence due to the physical, emotional and financial burden it places on patients and their families, leading to feelings of hopelessness, depression and frustration, which can cause patients to abandon treatment.

#### Side effects of TB medicines

Participants shared that some patients are reluctant to adhere to medication regimens due to concerns about the side effects of TB medicines. They mentioned side effects like difficulty in digestion, shivering, weakness and fatigue. They believed that they felt too weak after the infection, and it was difficult for them to digest such a heavy dosage, especially on an empty stomach.

These medicines seem harsh …(and) high in dosage. It puts a burden on my stomach. I get irritated, and my whole body starts trembling. When I take them, I cannot even stand up*.* (IDI-Haripur-Pak-Male-Patient)

After taking medicine, my body starts shivering, … and I want to cry*.* (IDI-Haripur-Pak-Male-Patient)

Carers also stated that patients usually lose their appetite due to constantly taking medicine, which causes physical weakness in their patients, leading to a reluctance among them to take medicines.

#### Depression and other comorbidities

The study found that a substantial number of patients with TB demonstrated symptoms of depression and other mental health conditions, which, in turn, affected their treatment adherence. Participants explained that due to the longer duration of TB treatment, heavy doses and stigma, many patients with TB get disheartened, lose hope about their recovery and end up suffering from depression. Healthcare providers explained that depression and other comorbidities reduce adherence to TB treatment as taking multiple medications for different conditions at the right time and in the correct doses can be overwhelming and confusing, and managing the complications of the comorbid condition can be physically and emotionally taxing. This can lead to fatigue, frustration and a decreased motivation to adhere to the TB treatment.

Yes, TB tablets are huge; patients get apprehensive about swallowing and digesting them. … the treatment is not for days; it is for months. …, using this tablet can cause stomach disorders, the colour of urine gets red, and it can also cause vomiting. Considering all these factors, the patient gets worried about the side effects of TB tablets, which can lead to depression*.* (IDI-Peshawar-Health Professional)

Depressive thoughts might decrease the will to recover among patients. In Peshawar, where Pashto is the commonly spoken language, people often use a genre of folk poetry called *‘tappa’* to express intense feelings. An apparently depressed patient used the following *tappa*, which reveals his anguish, hopelessness and despair.

Pa doctorano pora nashwa

Os ba maray wartha pa khpala ghazaoma

Translation: Doctors could not treat my illness; Now, I have no choice but to embrace death willy-nilly. (FGD-Peshawar-Pak-Male-Patient)

This tapa reveals the extreme hopelessness and disappointment of the patients. However, there is no mental health intervention currently at the DOTS centres to treat comorbid depression among patients with TB. The healthcare providers acknowledged that patients should be provided with psychosocial counselling, which is absent in the current treatment procedure.

Actually, it (counselling sessions) should be provided at a facility rather than at …… It should be bound to facility and educate them that it will cure their TB and depression. (FGD-Peshawar-Health Professional)

In short, the study highlights the complex interplay between physical and mental health in patients with TB, where the physical symptoms of TB can contribute to mental health issues and vice versa, which negatively impact their treatment adherence. This finding highlights the need for integrated mental health support and psychosocial counselling within TB care services to address the complex interplay between physical and mental health in patients with TB. The fact that healthcare providers acknowledge the need for psychosocial counselling but highlight its absence in the current treatment procedure underscores the need for policy and programme changes to address the mental health needs of patients with TB.

## Discussion

Our findings elucidate the views of patients, carers and healthcare professionals about the various barriers to treatment adherence among patients with TB. On a personal level, non-adherence can stem from the lack of awareness and knowledge about the importance of therapy completion, explanatory models of TB as a divine affliction and reliance on traditional and spiritual healing, and the old age and physical frailty of patients. The stigma associated with TB forces patients and carers to hide their illness, increasing the chances of treatment default. Lack of social support from family members and patriarchal cultures restrict female patients from visiting DOTS centres when required. Poverty, as a significant driver of health disparities, also plays a pivotal role in treatment adherence among poor patients who cannot regularly visit DOTS centres due to the country’s high transportation costs. Loss of job or decreased ability of patients with TB to work directly affects their adherence to the prescribed medication and diet plan. Previous studies have also identified that the socioeconomic status of patients, distance to healthcare, travelling time and nature of transport services significantly affect patient compliance with TB therapy.^29^

A key finding of our study was the substantial impact of gender on treatment adherence, particularly among women. The patriarchal norms in the region restrict women’s mobility, requiring them to be accompanied by male relatives when travelling outside the home. This often resulted in delays in seeking care, missing doses or skipping follow-up appointments. Similar gendered barriers to TB care have been reported in studies from other conservative societies, where women’s healthcare access is often contingent on male family members’ availability and approval.^30^ Interestingly, findings from a study conducted in the Philippines present a contrasting perspective on gender and TB treatment adherence. The study reported that men faced greater difficulties in accessing TB resources and services compared with women. This suggests that gendered health-seeking behaviours and barriers to care are context-dependent. While in Pakistan and among Afghan refugees, women struggle more due to mobility restrictions and caregiving responsibilities.^31^

The prolonged duration of TB treatment poses significant financial, physical and emotional challenges for patients and their families. Frequent visits to DOTS centres lead to transportation costs, lost wages and increased stress. Many patients experience hopelessness due to the slow recovery process, resulting in frustration and, in some cases, depression. These findings are in keeping with the existing literature.^32^ Addressing these gaps requires government intervention to enhance TB programme accessibility. Strengthening healthcare policies to make TB services more geographically and financially accessible is crucial for improving treatment adherence and health outcomes.

The current study underscores the high prevalence of depression among patients with TB and its significant impact on treatment adherence. The prevalence of depression among patients with TB is a well-established finding, with 48.9% of individuals receiving TB treatment suffering from depression, making it a major contributor to treatment default.^33 34^ For instance, a study conducted in rural Delhi highlighted the high burden of depression (nearly half of patients with TB), emphasising its role in poor treatment adherence.^35^ Despite its high burden, healthcare providers, as well as carers, were found to be least interested in the management of depression among patients with TB unless they were explicitly probed. The TB-depression comorbidity has been discussed elsewhere in a separate article, in which we have highlighted the need to develop and test culturally adaptive interventions for improving mental health outcomes and enhancing treatment adherence in TB.^36^

Older patients face significant challenges in adhering to TB treatment due to multimorbidity and physical frailty. The presence of comorbid conditions further complicates treatment, increasing the risk of default. Similar findings have been reported in other studies, suggesting that frailty and multiple chronic conditions among older patients with TB contribute to poorer treatment outcomes.^37^

Afghan nationals living in Pakistan face additional barriers in getting TB treatment as some of them frequently travel across the two countries and within Pakistan, and some do not possess valid registration documents as refugees, due to which they find it difficult to get access to DOTS centres in Pakistan. The lack of a Proof of Registration Card (POR Card) or other identification documents makes it difficult for Afghan refugees to access TB treatment in Pakistan. This bureaucratic hurdle can lead to delays in diagnosis, treatment initiation and adherence. Afghan refugees also face challenges due to their mobile lifestyle, which can disrupt their treatment regimen, leading to classification as ‘defaulters” and compromising their health outcomes. In addition, Afghan patients who are more economically vulnerable are affected more by transportation and medication expenses, which can potentially lead to delayed diagnosis, inadequate treatment and poor health outcomes.

The experience of Afghan patients in accessing TB treatment in Pakistan reflects the broader issues of statelessness and marginalisation faced by this population. Afghan refugees in Pakistan often lack access to basic rights and services, including healthcare, education and employment opportunities. The absence of documentation, such as the POR Card, can further marginalise this population, making it difficult for them to access essential services.

These findings related to refugees resonate with the findings of a study conducted in China, where the treatment outcomes among migrant patients were much worse when compared with the local patients because of the migrants’ poor education, low awareness of TB and poor financial conditions.^37^ Similarly, in many countries like Iran, for instance, Afghan refugees have access to services depending on their legal status, that is, having an immigration card, passport and visa or being undocumented. Many unregistered Afghans in Iran avoid going to public healthcare centres due to fear of deportation.^38 39^ The current study found that Afghan nationals were more inclined towards traditional healing practices, which is consistent with a study about Syrian refugees in the UK who became ‘their own doctor’ by turning to faith, rituals and nature for healing and comfort.^40^ This behaviour of refugees can be the result of their insecure and powerless position in the host society caused by a lack of resources, bargaining power and agency for collective action. The challenges faced by Afghan patients in accessing TB treatment in Pakistan highlight the need for healthcare policies and practices that are more inclusive and responsive to the needs of marginalised populations. Efforts are underway to address the issue of cross-border relocation of Afghan refugees to strengthen collaboration and information sharing among Afghanistan, Iran and Pakistan, to ensure continuity of care for Afghan refugee patients relocating from one country to another. As part of this work, a cross-border digital platform has been established to record and report TB cases among refugees, returnees and mobile populations.^41^

While this study focused on patients with TB in Pakistan and Afghan refugees residing there, many of the barriers to treatment adherence identified are not unique to this cultural or geographical context. Globally, patients with TB, particularly those from low-resource settings, face similar challenges such as poverty, limited transportation, stigma, prolonged treatment period, side effects of medication, mental and physical health comorbidities and gender-based inequalities in healthcare access. These are structural and psychosocial barriers that transcend national boundaries. For example, studies in Ghana and South Africa have highlighted the impact of poverty, transport costs and health literacy on treatment adherence.^12^ Similarly, the emotional toll of TB, particularly depression, has been consistently associated with poor treatment outcomes across various settings, including China, India and Ethiopia.^17 18 35^ Gender-related challenges, especially for women in patriarchal societies, are also widely reported across TB research globally (31). The findings from this study therefore offer transferable insights relevant for improving adherence in other high-burden TB settings, particularly for vulnerable and mobile populations.

This study has several limitations. It was conducted in only two districts of KP, Peshawar and Haripur, which may limit the generalisability of findings to other regions of Pakistan and Afghanistan. Participants were recruited from DOTS centres, which may have introduced selection bias by excluding individuals who had fully disengaged from care. Moreover, conducting interviews within healthcare settings may have influenced participants’ responses due to the formal environment, perceived authority of healthcare providers or concerns about confidentiality, potentially limiting the openness with which negative experiences or criticisms were shared. The study also relied on self-reported data, which is susceptible to recall and social desirability biases, particularly on sensitive issues such as stigma, mental health or gender-based barriers. Patients who had defaulted completely or dropped out of treatment were more difficult to access and were likely under-represented. Furthermore, the study focused on identifying the most prevalent and recurring themes across the three participant groups (patients, carers and healthcare providers), a comparative analysis of similarities and differences between these groups could have improved the findings.

This study uniquely explores TB treatment adherence in both Pakistani patients and Afghan refugees, offering a deeper understanding of how displacement, legal status and healthcare access intersect with adherence challenges. The political unrest and structural fragility in both Pakistan and Afghanistan further compound these issues. Addressing non-adherence requires multilevel, actionable strategies. We recommend integrating mental health support into TB services, particularly screening and brief interventions for depression, by leveraging scalable models such as the WHO’s mental health Gap Action Programme and task-shifting approaches that train TB health workers in basic psychosocial care (eg, behavioural activation, problem-solving therapy, motivational interviewing).^42^ Culturally tailored health education, gender-sensitive service delivery (eg, home-based or flexible follow-up options) and mobile tracking mechanisms for displaced or undocumented Afghan patients should also be prioritised. Financial and logistical support for transportation, diagnostics and nutrition would alleviate key structural barriers. Future research should focus on evaluating the effectiveness of integrated mental health and community-based adherence interventions, exploring healthcare provider behaviour and system-level gaps to improve long-term treatment continuity, particularly for mobile and refugee populations.

## Conclusion

Increasing treatment adherence requires a comprehensive approach to sensitise patients about the importance of treatment adherence, enabling and empowering patients to manage social stigma and enhance their decision-making power. Several personal, social, cultural and treatment-related barriers contribute to low treatment adherence among patients with TB. The legal status of Afghan nationals living in Pakistan, their frequent relocations and their low bargaining power and agency restrict their access to healthcare, which contributes to their treatment non-adherence. Among these factors, mental health challenges, particularly anxiety and depression, emerged as especially impactful across participant groups, often compounding other barriers such as stigma, isolation and economic stress. At the moment, there is no treatment or counselling available for mental health conditions at the DOTS level, warranting the need for mental health interventions that could improve treatment outcomes for both TB and depression.

## Supplementary material

10.1136/bmjopen-2025-100882 online supplemental file 1

## Data Availability

Data are available upon reasonable request.
