# Supplementary material for: Barriers to treatment adherence among patients with tuberculosis: a qualitative study of Pakistani nationals and Afghan refugees
Source: BMJ Open. 2025 Oct 5;15(10):e100882. doi: 10.1136/bmjopen-2025-100882 (PMC12506032; doi:10.1136/bmjopen-2025-100882)
Supplement: online supplemental file 1 [file bmjopen-15-10-s001.docx]

**Interview Procedure**

The interviews and focus group discussions (FGDs) were conducted between January and March 2023 in two districts of Khyber Pakhtunkhwa province, Pakistan, Peshawar and Haripur. The interviews were conducted by members of the qualitative research team, primarily trained in public health and qualitative research methods. These researchers had no prior relationship with the study participants. They were provided with background knowledge of tuberculosis (TB), including its prevention, treatment, and socio-cultural dimensions, to support informed and unbiased data collection.

The research assistants received professional training on qualitative interviewing skills, ethical considerations (including autonomy, beneficence, and non-maleficence), and managing discussions on sensitive topics such as stigma and mental health. They conducted 29 in-depth interviews (IDIs) and 11 FGDs with TB patients, their carers, and healthcare providers. Separate topic guides were developed for each participant group, translated into Urdu and Pashto, and pilot-tested before data collection began. Each FGD lasted between 50–70 minutes and each IDI lasted 35–45 minutes. All sessions were conducted in the preferred local language of participants and were audio-recorded with prior consent.

Participants were purposively selected to ensure diversity in gender, age, nationality (Pakistani and Afghan), and professional background. All participants provided written informed consent. To offset transportation costs and ensure equitable participation, each participant received a reimbursement of PKR 2000, which was reviewed and approved by the ethical committees.

**Reflexivity (Positionality) Statement**

Reflexivity is a core principle in qualitative research, emphasising transparency about the researcher's background, beliefs, and potential influence on the research process and interpretation. The study team acknowledged the socio-political and cultural sensitivities around TB, mental health, and refugee status in the region. Lead authors NS and FA are seasoned qualitative researchers with academic backgrounds in sociology and public health. Their expertise brought depth to the design, field engagement, and interpretation of findings, while maintaining a critical awareness of their positionality.

The primary researchers conducting the fieldwork were public health professionals based in Pakistan, familiar with the socio-cultural landscape but not personally affected by TB. This position provided both advantages (cultural and linguistic familiarity) and challenges (ensuring neutrality and avoiding assumptions). Special attention was paid to maintaining neutrality and empathy, particularly while engaging Afghan refugee participants, many of whom shared narratives of displacement, stigma, and marginalisation.

Given the high prevalence of stigma around TB and mental health, the team took care to conduct interviews and FGDs in settings that ensured privacy and psychological comfort. Female participants were interviewed by female researchers to respect cultural norms and encourage open sharing. The team recognised the inherent power dynamics between researchers and participants, especially when interacting with vulnerable groups like refugees and women. To minimise this, the research team emphasized rapport-building, active listening, and using local dialects to create a sense of familiarity and trust. Researchers consistently reflected on their role during data collection and analysis to ensure the authenticity of participant voices and guard against interpretative bias.
