# Supplementary material for: Barriers to treatment adherence among patients with tuberculosis: a qualitative study of Pakistani nationals and Afghan refugees
Source: BMJ Open. 2025 Oct 5;15(10):e100882. doi: 10.1136/bmjopen-2025-100882 (PMC12506032; doi:10.1136/bmjopen-2025-100882)
Supplement: Supplementary file 2 [file bmjopen-2025-100882.reviewer_comments.pdf]

PEER REVIEW HISTORY

BMJ Open publishes all reviews undertaken for accepted manuscripts. Reviewers are asked to complete a checklist review form and are provided with free text boxes to elaborate on their assessment. These free text comments are reproduced below.

ARTICLE DETAILS

Title (Provisional)

Barriers to treatment adherence among Tuberculosis patients: A qualitative study in Pakistan and Afghan refugee populations

Authors

Sanauddin, Noor; Ahmad, Fayaz; Rahim, Maryiam; Paracha , Muhammad Awais; khan, Zohaib; Qazi, Fatima Khalid; Rasool, Shaista; Butt, Mirrat; Naeem, Farooq; Khan, Muhammad Firaz; Sheikh, Saima; Kibria, Zeeshan; Fonseka, Nishani; Ul-Haq, Mian; Farooq, Saeed

---

VERSION 1 - REVIEW

**Reviewer**	**1**
**Name**	**Shaikh, Babar**
**Affiliation**	**Health Services Academy, School of Public health**
**Date**	**02-May-2025**
**COI**	**None**

This manuscript presents a well-designed qualitative study exploring the multifaceted barriers to tuberculosis (TB) treatment adherence among Pakistani and Afghan populations in the Khyber Pakhtunkhwa (KP) region of Pakistan. It incorporates insights from patients, carers, and healthcare providers using a combination of in-depth interviews and focus group discussions. The study highlights the social, cultural, emotional, and structural determinants of treatment nonadherence, with special attention to refugee-related challenges and mental health. The study offers actionable insights into how TB programs can be improved—particularly around mental health, gender-sensitive access, and refugee care.

However, I would like to draw the attention of the authors to the following weaknesses:

-There are several grammatical inconsistencies and typographical issues (e.g., missing conjunctions, misused articles, unclear phrases).

-The manuscript would benefit from professional copyediting to improve readability and precision, especially in the results and discussion sections.

-The “emotional” aspect of adherence, though mentioned frequently, could be better conceptualized. A brief theoretical framing (e.g., health belief model, syndemic theory) could strengthen interpretation.

-The authors mention incentives for participants. It would be appropriate to briefly justify this ethically in the methodology, especially with vulnerable groups (e.g., refugees).

-While legal barriers for Afghan patients are discussed, further elaboration on how registration documents impact access to TB care at institutional levels (e.g., referral, diagnostic follow-up) would add value.

-The manuscript does not clearly state how or when data saturation was achieved. Including this information would strengthen methodological transparency. Triangulation is mentioned, but there's no explanation of how contradictory views were handled or how analytical validity was ensured.

-The findings are descriptively rich but analytically shallow. The analysis tends to list themes without adequately interrogating patterns, contradictions, or power dynamics. For instance, gender is discussed superficially without deeper analysis of how patriarchy intersects with health system structure. Similarly, refugee status is treated as a fixed barrier rather than being linked to broader issues of statelessness or marginalization. The result is a missed opportunity to produce a more nuanced analysis.

-The manuscript is overloaded with long, sometimes repetitive quotes that are allowed to carry the narrative. This detracts from critical synthesis. Several quotes confirm rather than illuminate findings and should be reduced or replaced with stronger analysis.

-The study repeatedly emphasizes the need for psychosocial support but stops short of suggesting feasible intervention models. Even a brief mention of existing TB+mental health models (e.g., WHO mhGAP) or local pilot efforts would enhance the discussion.

-Table 2 is detailed but could be reformatted for clarity. Consider aggregating some rows for better visual impact.

-Consider explicitly acknowledging the potential limitations of conducting research in DOTS centres (e.g., selection bias, influence of setting).

-Use consistent terms when referring to TB types (e.g., DR-TB, MDR-TB) and participant groups.

With these suggested revisions for clarity, structure, and theoretical integration, it may make a worthwhile contribution to the readership of the journal and the global TB control discourse.

**Reviewer**	**2**
**Name**	**Lustrea, Anca**
**Affiliation**	**West University of Timisoara, of Educational Sciences**

**Date**	**06-Jul-2025**
**COI**	**None**

The article addresses a very important topic in the context of the management of TB patients, the in-depth identification of factors that determine treatment adherence.

I recommend that the article be accepted with minor revisions. My reason for this recommendation is that the presented research is methodically correct and presents the research and its results in a comprehensive, structured scientific manner.

However, I propose to the authors' attention some recommendations for revision.

• First of all, the categories of factors taken into account and those determined by the analysis of the results can create confusion. The categories of factors, defined in the title are - social, cultural and emotional factors, in the objectives they are presented as personal, cultural, and context-specific barriers and the categories of determined factors presented in the abstract are socio-cultural, patient-related, Afghan refugees-specific factors and treatment-related. I recommend making the categories of factors compatible for better coherence of the research approach.

• Also, to clarify and standardize whether they are factors (seen as potentially having a positive or negative determination on TB treatment) or barriers (interpreted with a negative determination only), as presented in the objectives.

• In the abstract, the main factor presented in the conclusions section, namely anxiety, is not found in the factors presented in the abstract in the results section. Conclusions must follow directly from the results.

• I recommend presenting a research gap right from the abstract.

• I recommend moving the Socio-demographic characteristics of study participants section from results to method, participants.

• It is not understood why the cumulative data in table 1 and table 2 do not coincide. I recommend to insert an explanation.

• In the Data analysis section it is stated `Finally, we triangulated the key findings from the three participant groups, exploring their similarities and differences`, but nowhere do comparisons between the responses of the three groups, similarities and differences between their perceptions appear. It would be interesting to address these differences and similarities in the results section, for each identified theme.

• I recommend introducing a section of practical recommendations and future directions for research.

• I recommend emphasizing some general characteristics, independent of the cultural context, that can be taken over by readers from other cultures/geographical areas.

• I recommend introducing a limits section, although they are briefly presented at the beginning, where they should be presented more precisely.

• The presentation of anxiety and depression as the main factor in the conclusions, although no hierarchy of these factors was made in the results section, requires a justification for placing mental health factors at the forefront of the discussion.

Congratulations on your research!

VERSION 1 - AUTHOR RESPONSE

Reviewer: 1
Prof. Babar Shaikh, Health Services Academy
Comments to the Author:

This manuscript presents a well-designed qualitative study exploring the multifaceted barriers to tuberculosis (TB) treatment adherence among Pakistani and Afghan populations in the Khyber Pakhtunkhwa (KP) region of Pakistan. It incorporates insights from patients, carers, and healthcare providers using a combination of in-depth interviews and focus group discussions. The study highlights the social, cultural, emotional, and structural determinants of treatment nonadherence, with special attention to refugee-related challenges and mental health. The study offers actionable insights into how TB programs can be improved—particularly around mental health, gender-sensitive access, and refugee care.

1	There are several grammatical inconsistencies and typographical issues (e.g., missing conjunctions, misused articles, unclear phrases). The manuscript would benefit from professional copyediting to improve readability and precision, especially in the results and discussion sections.	Response: We appreciate the reviewer's helpful suggestion. We have carefully reviewed the manuscript and performed thorough copyediting to address grammatical inconsistencies, typographical issues, and unclear phrasing—particularly in the results and discussion sections. We believe these revisions have improved the clarity and overall readability of the manuscript.
2	The "emotional" aspect of adherence, though mentioned frequently, could be better conceptualized. A brief theoretical framing (e.g., health belief model, syndemic theory) could strengthen interpretation.	Response: While we acknowledge the potential value of theoretical models such as the Health Belief Model or syndemic theory in structuring analyses of treatment behaviour, we intentionally chose an inductive qualitative approach that prioritized participants' lived experiences and cultural interpretations of TB adherence. Applying a pre-existing theoretical framework at the outset risked imposing external constructs that might have constrained the emergent emotional, social, and contextual nuances revealed in our data, such as the

		use of Pashto poetry to express despair, or culturally specific forms of stigma and resistance. Instead, our approach allowed us to capture complex, grounded insights into how emotional distress intersects with poverty, gender norms, and displacement. While our findings may align with aspects of behavioural or syndemic models, we believe the strength of our study lies in letting theory emerge from the data, rather than fitting data to theory.
3	The authors **mention incentives for participants.** It would be appropriate to briefly justify this ethically in the methodology, especially with vulnerable groups (e.g., refugees).	Response: Thank you for this valuable comment. We agree that ethical justification is particularly important when working with vulnerable populations such as refugees. We have now clarified this in the methodology section by explicitly stating that the financial reimbursement provided to participants was strictly for travel costs and intended to reduce economic barriers to participation. We have added the following text in the methods section of the manuscript:<br><br>*"Reimbursement for travel costs incurred in attending the interview and focus group discussions (FGDs) at local health facilities. This amount was determined based on average local transport expenses to ensure that participation in the study did not place a financial burden on the individuals involved. This incentive aimed to reduce financial barriers that might otherwise prevent participation, particularly among individuals from socioeconomically disadvantaged backgrounds. The amount was carefully determined to avoid any form of coercion or undue influence and was approved by the ethics committee (Keele and KMU) as part of the study protocol"*.
4	While legal barriers for Afghan patients are discussed, further **elaboration on how registration documents impact access to TB care at institutional levels (e.g., referral, diagnostic follow-up) would add value.**	Response: Thank you for this insightful comment. We agree that further elaboration on the role of registration documents in shaping institutional access to TB care for Afghan patients adds important depth to our analysis. We have now expanded the relevant section in the manuscript to clarify how the absence of legal identity documents such as Proof of Registration (PoR) or Afghan Citizen Cards (ACC) restricts Afghan patients' access to diagnostic services, formal enrolment in TB programs, and continuity of follow-up care. We have also described how undocumented status contributes to delays, repeated visits, and inconsistent treatment, often resulting in patients being labelled as 'defaulters'. The expanded section now discusses the implications of frequent displacement and administrative exclusion on patient-provider relationships, case tracking, and treatment outcomes. These revisions aim to strengthen the institutional-level understanding of how legal and mobility-related vulnerabilities undermine TB care access for refugee populations. Response in the manuscript:<br><br>"The lack of a Proof of Registration Card (POR Card) or other identification documents makes it difficult for Afghan refugees to access TB treatment in Pakistan. This bureaucratic hurdle can lead to delays in diagnosis, treatment initiation, and adherence. Afghan refugees also face challenges due to their mobile lifestyle, which can

		disrupt their treatment regimen, leading to classification as "defaulters" and compromising their health outcomes. In addition, Afghan patients who are more economically vulnerable are affected more by transportation and medication expenses, which can potentially lead to delayed diagnosis, inadequate treatment, and poor health outcomes.<br>The experience of Afghan patients in accessing TB treatment in Pakistan reflects the broader issues of statelessness and marginalization faced by this population. Afghan refugees in Pakistan often lack access to basic rights and services, including healthcare, education, and employment opportunities. The absence of documentation, such as the POR Card, can further marginalize this population, making it difficult for them to access essential services".
5	The manuscript does not clearly state how or when data saturation was achieved. Including this information would strengthen methodological transparency. Triangulation is mentioned, but there's no explanation of how contradictory views were handled or how analytical validity was ensured.	RESPONSE: We appreciate the reviewer's observation regarding the determination of data saturation. We have added the following paragraph to explain how data saturation was determined in this study. We have added:<br><br>*"Throughout the data collection, the research team engaged in daily debriefing sessions with the data collection team, wherein we discussed emerging themes, methodological and practical challenges, and the necessity for additional data collection. These iterative sessions enabled us to reflectively assess our recruitment strategy and determine whether further interviews or focus groups would yield novel insights. Given the substantial sample size of this study, our team collectively determined that continued data collection would likely result in diminishing returns, thereby indicating saturation. Consequently, we ceased data collection, confident that our dataset was sufficiently comprehensive to address our research objectives."*<br><br>Regarding Triangulation, we have added the following paragraph in the manuscripts.<br><br>*"To ensure analytical validity, we employed multiple coders to independently code the data. We also engaged in peer debriefing, where colleagues reviewed and provided feedback on our analysis, to identify potential biases and improve the validity of our findings. These steps helped to increase our confidence in the accuracy and reliability of our results. In addition, to enhance the validity and reliability of our findings, we utilized a data triangulation approach, wherein multiple data sources were leveraged to corroborate and validate our results. Specifically, we integrated data from interviews and focus groups to facilitate a comprehensive understanding of the phenomenon under investigation. The triangulation of data sources enabled us to verify the consistency of our findings across different methodological approaches, thereby augmenting the accuracy and reliability of our results. Furthermore, this approach allowed us to capture*

		a more nuanced and multifaceted understanding of the phenomenon, thereby enriching our insights. In instances where divergent perspectives emerged, we employed negative case analysis to scrutinize these discrepancies and elucidate the underlying factors contributing to these contradictions. This analytical strategy enabled us to refine our understanding and provide a more robust interpretation of the data."
6	The findings are descriptively rich but analytically shallow. The analysis tends to list themes without adequately interrogating patterns, contradictions, or power dynamics. For instance, gender is discussed superficially without deeper analysis of how patriarchy intersects with health system structure. Similarly, refugee status is treated as a fixed barrier rather than being linked to broader issues of statelessness or marginalization. The result is a missed opportunity to produce a more nuanced analysis.	RESPONSE: We wholeheartedly agree with the reviewer's comment. In response, we have revisited the themes and expanded upon the analytical rigor wherever possible, while mindful of the article's length constraints. To further elucidate the complexities surrounding each theme and its policy implications, we have incorporated additional paragraphs across most themes and sub-themes. These additions have been reproduced below.<br><br>***Lack of knowledge about TB and its treatment:***<br>*"The quotes from healthcare professionals and carers underscore the need for improved patient education, awareness, and support to promote treatment adherence and address the challenges posed by misconceptions and cultural beliefs about TB."... "The lack of knowledge and awareness about TB and its treatment among patients and carers is a significant barrier to treatment adherence, with patients often taking medicines based on perceived wellness rather than following doctor's advice, leading to drug resistance. Additionally, some patients' limited trust in TB medicines, cultural beliefs (e.g., TB being a divine affliction), and refusal to take preventive measures further contribute to delayed registration at DOTS and non-adherence to medication."*<br><br>***Multimorbidity and frailty in old age patients***<br>*"Older patients with TB face unique challenges, including physical frailty, co-morbid conditions, and limited understanding of their disease, which can contribute to treatment failure and default. The role of family support is critical in helping older patients adhere to medication and treatment regimens, particularly when patients experience cognitive or physical limitations that affect their ability to manage their treatment. By acknowledging these factors, healthcare providers can develop targeted strategies to support older patients and improve their treatment outcomes."*<br><br>***Stigma:***<br>*"The stigma surrounding TB is deeply rooted in cultural and social norms which leads to social isolation, mental health deterioration, and poor treatment adherence. The quotes from patients highlight the emotional pain and distress caused by stigma, which can be as debilitating as the physical symptoms of the disease. The stigma surrounding TB is not just a personal issue but also a public health concern, as it can lead to poor treatment adherence and increased transmission of the disease. By addressing TB-related stigma, we can improve treatment*

			adherence, reduce social isolation, and promote the overall well-being of patients with TB."<br><br>***Gender:***<br>*The quote from the health professional highlights the lack of support from family members, particularly husbands, in recognizing and addressing women's health needs. This can lead to women feeling isolated, unsupported, and struggling to manage their condition. Women patients find it more challenging to follow treatment regimens because of cultural factors limiting their decision-making powers about health and wellbeing. The requirement for women to be accompanied by male relatives when going out of the home can significantly delay their access to healthcare services, including TB treatment. Similarly, women patients' responsibility to take care of children and dependents can make it challenging for them to prioritize their own health needs. This can lead to missed appointments, delayed diagnosis, and poor treatment adherence. The widespread belief that women use illness as an excuse to avoid household chores or seek attention can contribute to the stigma and lack of understanding around women's health issues. In short, the findings of this study highlighted the power dynamics at play in patriarchal society, where women's decision-making powers about their health and wellbeing are limited. This can lead to women being unable to prioritize their own health needs, adhere to treatment regimens, or seek healthcare services without permission from their male family members.*<br><br>***Refugees-***<br>*"The lack of a Proof of Registration Card (POR Card) or other identification documents makes it difficult for Afghan refugees to access TB treatment in Pakistan. This bureaucratic hurdle can lead to delays in diagnosis, treatment initiation, and adherence. Afghan refugees also face challenges due to their mobile lifestyle, which can disrupt their treatment regimen, leading to classification as "defaulters" and compromising their health outcomes. In addition, Afghan patients who are more economically vulnerable are affected more by transportation and medication expenses, which can potentially lead to delayed diagnosis, inadequate treatment, and poor health outcomes.*<br>*The experience of Afghan patients in accessing TB treatment in Pakistan reflects the broader issues of statelessness and marginalization faced by this population. Afghan refugees in Pakistan often lack access to basic rights and services, including healthcare, education, and employment opportunities. The absence of documentation, such as the POR Card, can further marginalize this population, making it difficult for them to access essential services."*<br>*In the discussion section of the article, we have added this sentence of implication of this finding:*

		"The challenges faced by Afghan patients in accessing TB treatment in Pakistan highlight the need for healthcare policies and practices that are more inclusive and responsive to the needs of marginalized populations."<br><br>***Traditional Medicines:***<br>*"The reliance on traditional medicine can lead to delayed diagnosis and treatment of TB. Patients may try various traditional remedies before seeking medical attention from a healthcare provider, which can worsen their condition and reduce the effectiveness of treatment. … The reliance on traditional medicine among patients in Pakistan and Afghanistan reflects the complex interplay between cultural, social, and economic factors that influence treatment-seeking behavior. Some potential factors contributing to this reliance include limited access to healthcare, leading patients to seek alternative forms of treatment. Traditional medicine is deeply ingrained in the cultural and social fabric of these countries, making it a preferred option for many patients. Lastly, patients may not be aware of the effectiveness of modern medicine in treating TB, or they may not understand the risks associated with traditional remedies."*<br><br>***Depression and other co-morbidity in TB***<br>*"The study highlights the complex interplay between physical and mental health in TB patients, where the physical symptoms of TB can contribute to mental health issues, and vice versa, which negatively impact their treatment adherence. This finding highlights the need for integrated mental health support and psychosocial counselling within TB care services to address the complex interplay between physical and mental health in TB patients. The fact that healthcare providers acknowledge the need for psychosocial counselling but highlight its absence in the current treatment procedure underscores the need for policy and programmatic changes to address the mental health needs of TB patients".*
8	The study repeatedly emphasizes the need for psychosocial support but stops short of suggesting feasible intervention models. Even a brief mention of existing TB+mental health models (e.g., WHO mhGAP) or local pilot efforts would enhance the discussion.	Response: Thank you for this constructive suggestion. We have now expanded the discussion to include feasible and scalable intervention models that address the psychosocial needs of TB patients.<br><br>*"Addressing non-adherence requires multi-level, actionable strategies. We recommend integrating mental health support into TB services, particularly screening and brief interventions for depression, by leveraging scalable models such as the WHO's mental health Gap Action Programme (mhGAP) and task-shifting approaches that train TB health workers in basic psychosocial care (e.g., behavioural activation, problem-solving therapy, motivational interviewing)".*

9	Table 2 is detailed but could be reformatted for clarity. Consider aggregating some rows for better visual impact.	Response: Thank you for the suggestion. We agree that visual clarity is important. However, given the qualitative nature of our study and the need to transparently present subgroup-level sampling across sites, we believe the current detailed format enhances clarity and interpretability. We have still split the table for visual clarity.
10	Consider explicitly acknowledging the potential limitations of conducting research in DOTS centres (e.g., selection bias, influence of setting).	Response: Thank you for this insightful comment. We have now explicitly acknowledged the potential limitations of conducting the research in DOTS centres, particularly in terms of possible selection bias and the influence of the setting on participants' responses.<br><br>*"This study has several limitations. It was conducted in only two districts of KP, Peshawar and Haripur, which may limit the generalisability of findings to other regions of Pakistan and Afghanistan. Participants were recruited from DOTS centres, which may have introduced selection bias by excluding individuals who had fully disengaged from care. Moreover, conducting interviews and focus groups within healthcare settings may have influenced participants' responses due to the formal environment, perceived authority of healthcare providers, or concerns about confidentiality, potentially limiting the openness with which negative experiences or criticisms were shared. The study also relied on self-reported data, which is susceptible to recall and social desirability biases, particularly on sensitive issues such as stigma, mental health, or gender-based barriers. Patients who had defaulted completely or dropped out of treatment were more difficult to access and are likely underrepresented. Furthermore, the study focused on identifying the most prevalent and recurring themes across the three participant groups (patients, carers, and healthcare providers), a comparative analysis of similarities and differences between these groups could have improved the findings.*
11	Use consistent terms when referring to TB types (e.g., DR-TB, MDR-TB) and participant groups.	Response: we have now used DR TB and DS TB instead MDR that was used only once

Reviewer: 2

Dr. Anca Lustrea, West University of Timisoara

Comments to the Author:

The article addresses a very important topic in the context of the management of TB patients, the in-depth identification of factors that determine treatment adherence.

I recommend that the article be accepted with minor revisions. My reason for this recommendation is that the presented research is methodically correct and presents the research and its results in a comprehensive, structured scientific manner.

However, I propose to the authors' attention some recommendations for revision.

12	First of all, the categories of factors taken into account and those determined by the analysis of the results can create confusion. The categories of factors, defined in the title are - social, cultural and emotional factors, in the objectives they are presented as personal, cultural, and context-specific barriers and the categories of determined factors presented in the abstract are socio-cultural, patient-related, Afghan refugees-specific factors and treatment-related. I recommend making the categories of factors compatible for better coherence of the research approach	Response: Thank you for this valuable observation. We agree that consistency in terminology is important for clarity. In response, we have revised the phrasing across the title, abstract, objectives, and results to ensure alignment in how the categories of barriers are described. We now consistently refer to: Patient-Related, Socio-cultural, andTreatment-Related barriers which better reflect both the study objectives and the thematic findings.
13	Also, to clarify and standardize whether they are factors (seen as potentially having a positive or negative determination on TB treatment) or barriers (interpreted with a negative determination only), as presented in the objectives.	RESPONSE: We agree with the reviewer's observation regarding the inconsistent use of terminology in our article. Specifically, we employed the terms 'factors' and 'barriers' interchangeably, which may have led to ambiguity and confusion for readers. In response to this feedback, we have meticulously revised the manuscript to ensure consistency in terminology. Throughout the document, we have opted to utilize the term 'barriers' to underscore the paper's focus on the challenges and obstacles that adversely impact adherence to tuberculosis treatment among patients.
14	In the abstract, the main factor presented in the conclusions section, namely anxiety, is not found in the factors presented in the abstract in the results section. Conclusions must follow directly from the results.	RESPONSE: We acknowledge the reviewer's observation regarding the prominence of depression in our article. Indeed, the theme of depression and its impact on individuals has been extensively explored throughout the manuscript. In addition to its mention in the results section, specifically under the subheadings of Stigma and Poverty, we have dedicated a distinct sub-theme (last sub-theme in the result section) to examining depression and other comorbidities as substantial barriers to adherence to tuberculosis treatment.
15	I recommend presenting a research gap right from the abstract	Response: Thank you for this valuable suggestion. We have revised the abstract to explicitly state the research gap at the beginning, highlighting the lack of qualitative research on socio-cultural and emotional barriers to TB treatment adherence, particularly among Afghan refugees in Pakistan.<br><br>*“Objectives:*<br>*Nonadherence to tuberculosis (TB) treatment remains a major challenge in high-burden regions, yet few studies have qualitatively examined the socio-cultural and emotional*

		barriers to adherence, particularly among Afghan refugees in Pakistan."
16	I recommend moving the Socio-demographic characteristics of study participants section from results to method, participants.	Response: Thank you for your thoughtful suggestion. We agree that socio-demographic details are crucial to understanding the context of the study. However, we have chosen to present the socio-demographic characteristics of study participants in the Results section to align with standard qualitative reporting practices, where participant characteristics are often presented at the start of the findings to contextualize the data and themes that follow. This placement helps readers interpret the analysis with reference to who said what, and under which demographic conditions. We hope the rationale for retaining it in the Results section is acceptable.
17	It is not understood why the cumulative data in table 1 and table 2 do not coincide. I recommend inserting an explanation.	Response: Table 1 counts groups (e.g., "7 FGDs" in Peshawar), while Table 2 counts individual participants within those groups (e.g., "46 people" in Peshawar FGDs). Subcategories (e.g., Afghan patients) were summed differently. Table 1 uses raw group counts, while Table 2 uses gender-split individuals. Nested categories (i.e., Peshawar/Haripur →IDIs/ FGDs Patients → Afghani /Pakistani → Male/Female") are hard to understand visually without charts. This is the limitation of using tables instead of charts/graphs, which is acknowledged here.<br>The mismatch falsely implies inconsistency when they simply reflect different scales (groups vs. individuals). The caption of Table 1 has been changed for more clarity. "Number of FGDs and IDIs Conducted by Participant Type and Nationality"<br>Table 1 also lacks a footnote to explain the relationship between the two tables. A footnote has been added below Table 1 "Note: Each FGD contained 6-7 participants (see Table 2)"<br>To enhance clarity Caption of Table 2, has also been also changed to "Demographics of individual participants within each IDI and FGDs"<br><br>We have now split table 2 for more visual clarity.
18	In the Data analysis section it is stated ` Finally, we triangulated the key findings from the three participant groups, exploring their similarities and differences` , but nowhere do comparisons between the responses of the three groups, similarities and differences between their perceptions appear. It would be interesting to address these differences and similarities in the results section, for each identified theme.	RESPONSE: We acknowledge that the reviewers have highlighted a significant limitation of our study. We have now explicitly acknowledged this limitation in the article. We have now explained the issue of triangulation in detail in the data analysis section of the article. However, while our analysis focused on identifying the most prevalent and recurring themes across the three participant groups (patients, carers, and healthcare providers), a comparative analysis of similarities and differences between these groups could have improved our findings. We have duly acknowledged this limitation in the discussion section, specifically in the limitation's subsection.

19	I recommend introducing a section of practical recommendations and future directions for research	Response: We appreciate the reviewer's valuable suggestion. In response, we have incorporated practical recommendations and future directions for research into the discussion section to ensure a more actionable conclusion to our findings.<br><br>*"Addressing non-adherence requires multi-level, actionable strategies. We recommend integrating mental health support into TB services, particularly screening and brief interventions for depression, by leveraging scalable models such as the WHO's mental health Gap Action Programme (mhGAP) and task-shifting approaches that train TB health workers in basic psychosocial care (e.g., behavioural activation, problem-solving therapy, motivational interviewing). Culturally tailored health education, gender-sensitive service delivery (e.g., home-based or flexible follow-up options), and mobile tracking mechanisms for displaced or undocumented Afghan patients should also be prioritised. Financial and logistical support for transportation, diagnostics, and nutrition would alleviate key structural barriers. Future research should focus on evaluating the effectiveness of integrated mental health and community-based adherence interventions, exploring healthcare provider behaviour and system-level gaps to improve long-term treatment continuity, particularly for mobile and refugee populations."*
20	I recommend emphasizing some general characteristics, independent of the cultural context, that can be taken over by readers from other cultures/geographical areas.	Response: We thank the reviewer for this thoughtful suggestion. In response, we have ensured that the discussion highlights broader themes, such as the impact of mental health, stigma, gender dynamics, and socioeconomic hardship, that may resonate across diverse cultural and geographical contexts.<br>*While this study focused on TB patients in Pakistan and Afghan refugees residing there, many of the barriers to treatment adherence identified are not unique to this cultural or geographic context. Globally, TB patients, particularly those from low-resource settings face similar challenges such as poverty, limited transportation, stigma, prolonged treatment period, side effects of medication, mental and physical health comorbidities, and gender-based inequalities in healthcare access. These are structural and psychosocial barriers that transcend national boundaries. For example, studies in Ghana and South Africa have highlighted the impact of poverty, transport costs, and health literacy on treatment adherence (12). Similarly, the emotional toll of*

		TB, particularly depression, has been consistently associated with poor treatment outcomes across various settings, including China, India, and Ethiopia (17,18, 34) Gender-related challenges, especially for women in patriarchal societies, are also widely reported across TB research globally (30) The findings from this study therefore offer transferable insights relevant for improving adherence in other high-burden TB settings, particularly for vulnerable and mobile populations."
21	I recommend introducing a limits section, although they are briefly presented at the beginning, where they should be presented more precisely.	Response: We thank the reviewer for this helpful suggestion. In response, we have added a new, detailed paragraph outlining the study's limitations within the discussion section, just before the final paragraph.<br><br>*"This study has several limitations. It was conducted in only two districts of KP, Peshawar and Haripur which may limit the generalisability of findings to other regions of Pakistan and Afghanistan. Participants were recruited from DOTS centres, which may have introduced selection bias by excluding individuals who had fully disengaged from care. Moreover, conducting interviews within healthcare settings may have influenced participants' responses due to the formal environment, perceived authority of healthcare providers, or concerns about confidentiality, potentially limiting the openness with which negative experiences or criticisms were shared. The study also relied on self-reported data, which is susceptible to recall and social desirability biases, particularly on sensitive issues such as stigma, mental health, or gender-based barriers. Patients who had defaulted completely or dropped out of treatment were more difficult to access and are likely underrepresented. Furthermore, the study focused on identifying the most prevalent and recurring themes across the three participant groups (patients, carers, and healthcare providers), a comparative analysis of similarities and differences between these groups could have improved the findings.*
22	The presentation of anxiety and depression as the main factor in the conclusions, although no hierarchy of these factors was made in the results section, requires a justification for placing mental health factors at the forefront of the discussion.	Response: We thank the reviewer for this important observation. While our study identified multiple personal, social, and structural barriers to TB treatment adherence, mental health, particularly anxiety and depression, emerged as a cross-cutting theme that exacerbated other barriers. As discussed in the manuscript, depression was not only prevalent but also found to affect patients' motivation, engagement, and perceived ability to complete treatment. To reflect this, we have revised the conclusion to clarify the rationale

		for highlighting mental health as a major factor without implying a formal hierarchy.<br><br>*"Increasing treatment adherence requires a comprehensive approach to sensitise patients about the importance of treatment adherence, enabling and empowering them to manage social stigma and enhance their decision-making power. Several personal, social, cultural, and emotional factors contribute to low treatment adherence among TB patients. The legal status of Afghan nationals living in Pakistan, their frequent relocations, and their low bargaining power and agency restrict their access to healthcare, which contributes to their treatment non-adherence. Among these factors, mental health challenges, particularly anxiety and depression, emerged as especially impactful across participant groups, often compounding other barriers such as stigma, isolation, and economic stress. At the moment, there is no treatment or counselling available for mental health conditions at the DOTS level, warranting the need for mental health interventions that could improve treatment outcomes for both TB and depression."*

VERSION 2 - REVIEW

**Reviewer**	**1**
**Name**	**Shaikh, Babar**
**Affiliation**	**Health Services Academy, School of Public health**
**Date**	**11-Aug-2025**
**COI**	

Authors have nicely answered all concerns and points raised in the previous review. Length o fthe paper is only concern left.

**Reviewer**	**2**
**Name**	**Lustrea, Anca**
**Affiliation**	**West University of Timisoara, of Educational Sciences**
**Date**	**19-Aug-2025**

COI

Congratulations for your research and time and effort put into it!

The article addresses a very important topic in the context of the management of TB patients and the factors that determine treatment adherence.

I recommend that the article be accepted in its current form. My reason for this recommendation is that the presented research is methodically correct in all aspects and presents the research and its results in a comprehensive, structured scientific manner.

The abstract concisely reports the aims and outcomes of the research. The methods, results, and conclusion are presented in a structured manner.

The theoretical framework is well written and introduces the research concepts in a comprehensive manner. All the presumptions and affirmations are supported by up-to-date references.

The method is described in detail. The research design is correct in all aspects. The assessment methods are valid for research purposes.

The results are presented in a structured manner and easy to understand.

The findings section offers in-depth interpretations of the results, from which valid conclusions are drawn.

Congratulations!
